# Lower Eyelid Dark Circles (Tear Trough and Lid-Cheek Junction): A Stepwise Assessment Framework

**DOI:** 10.1093/asj/sjae058

**Published:** 2024-03-15

**Authors:** Steven Liew, Simone Doreian, Wachira Kunathathorn, Stephanie Lam, Alvin Jorge, Lam Bee Lan, Ellen Selkon, Sean Arendse, Jodie Silleri, Tara Telfer

## Abstract

**Background:**

Despite increasing popularity, the use of hyaluronic acid (HA) fillers for the correction of dark under-eye shadows remains challenging. Specific guidance on patient assessment is limited.

**Objectives:**

The aim of this study was to develop a stepwise assessment framework for lower eyelid dark shadows to help practitioners classify patients based on their underlying problems and facilitate a more strategic approach to treatment.

**Methods:**

Literature review and peer collaboration informed the current availability of educational material for use by experienced injectors when assessing patients presenting with dark circles. A practitioner survey provided insight into current practices. A focus group convened to review the survey results and discuss best practice approaches to patient assessment.

**Results:**

Surveyed practitioners (*n* = 39) reported patient concern about under-eye hollows (91%), dark eye circles (80%), and looking tired (60%). All (100%) agreed that midcheek volume was critical when treating tear-trough depression, and only 26% reported use of a tear-trough classification system. The focus group developed a framework for assessing tear-trough depression and the lid-cheek junction in patients presenting with dark circles. Key factors within this framework included the importance of appropriate lighting when conducting a visual inspection, regional inspection of the cheek and tear trough, palpation of the orbital rim and soft tissues, determination of the orbital vector, and assessment of lower eyelid pigmentation and skin quality.

**Conclusions:**

Careful step-by-step assessment can reduce the challenges of treating dark circles by identifying patients in whom dark eye circles may be improved without the need to directly inject filler into the tear trough.

**Level of Evidence: 5:**

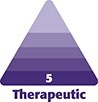

A youthful periocular region is characterized by fullness of the periorbital area, minimal or no skin excess, minimal pigmentation or skin wrinkles, and appropriate brow height and shape.^1^ By contrast, lower eyelid dark shadows are characterized by soft tissue loss and bony resorption, which create a sunken appearance and cast dark shadows despite adequate rest.^2^ The presence of dark shadows negatively influences perceptions of youthfulness, attractiveness, and emotion, often resulting in the utilization of descriptors such as tiredness, unhappiness, or sadness.^3^

The lower periorbital region is one of the most common areas for which patients seek minimally invasive facial aesthetic treatment, citing under-eye bags and dark circles as their primary concerns.^4^ The use of hyaluronic acid (HA) gel filler for the correction of dark under-eye shadows, with its quick results and minimal recovery time, has become a popular rejuvenation treatment and is associated with high levels of posttreatment patient satisfaction.^5^ Yet the lower periorbital is a challenging area to inject.^6,7^ Complications observed with dermal fillers to treat this area include mild, self-limiting adverse effects (puffiness, swelling, bruising), persistent irregularities (irregular surface contours, edema, and a bluish hue or Tyndall effect), and severe (irreversible vision loss) or long-term complications (granulomas).^8,9^ These unacceptable aesthetic outcomes can result from overcorrection, too superficial injections, and inappropriate choice of filler.^10^ Good knowledge of periorbital anatomy, product selection, and appropriate injection technique are therefore vital for optimal treatment outcomes. In recognition of this, the recent literature provides details of therapeutic approaches and comprehensive reviews of periorbital anatomy, as well as guidance for the recognition, prevention, and management of filler-associated complications that are most commonly encountered when treating this region.^6-8,10-16^ However, specific guidance on assessment is limited. This paper draws on recent concepts and current clinical practices to help guide the practitioner through an assessment framework for lower eyelid dark shadows that can be undertaken before establishing a treatment plan with the patient.^15,17^ The aim is to provide an assessment that will better enable the practitioner to classify the patient based on their underlying problems and facilitate a more strategic approach to treatment.

## METHODS

The focus of this work was on identifying key characteristics that contribute to patient dissatisfaction with the eye area and deriving an assessment framework to account for these characteristics.

This study was conducted in multiple stages and included multidisciplinary collaboration, a practitioner survey, a focus group, and real-world experience. The study sponsor selected 8 senior educators from the within the Galderma Aesthetics Injection Network (GAIN) and invited an additional 32 GAIN members from within the Asia Pacific region to participate in a scientific exchange to discuss and develop educational materials. The Scientific Exchange Working Group comprised 8 clinicians specializing in dermatology, cosmetic medicine, plastic surgery, and maxillofacial surgery. All of these practitioners provided their first and second choice options across 4 primary domains (perioral, profile, skin, and periorbital). Two members of the working group and 8 of the practitioners were then assigned to each domain, based on their preference and their experience. We discuss here the results from the periorbital domain.

A literature search was conducted with search terms constructed to provide relevant information regarding 3 core considerations: (1) anatomy of the periorbital region, (2) characterization of key features of the periorbital region and related assessment frameworks, and (3) practical application and choice of treatment for minimally invasive periorbital treatment. The working group convened to discuss the results of the literature search and their current clinical practices. The outputs from this multidisciplinary discussion were then utilized to develop a short practitioner survey (Appendix, available at www.aestheticsurgeryjournal.com) to assess the experience, perceptions, and opinions of a wider group of clinicians.

The practitioner survey was completed online though an emailed link. The survey responses were returned to a medical writer for deidentification, aggregation, and analysis. All working group members and invited physicians completed the survey, irrespective of which domain they had elected to focus on. The survey enabled practitioners to rank the importance of a number of characteristics that contribute to youthful eyes with an 11-point visual analog scale (0 = no contribution, 10 = most contribution). Invited practitioners completed the survey in February 2022, and data in the returned surveys were summarized descriptively. In March 2022 a hybrid focus group meeting was held, during which the authors reviewed the survey results, discussed best practice approaches to patient assessment, and developed an assessment framework.

## RESULTS

### Practitioner Survey Responses

The response rate to the practitioner survey was 98% (39/40 surveys returned). Respondents were from 18 countries (predominantly Australia 46%; Supplemental Table 1, available at www.aestheticsurgeryjournal.com). The majority were cosmetic physicians (61%), 21% were dermatologists and 18% were surgeons; almost all respondents (95%) had been practicing aesthetic medicine for more than 10 years. Respondents recognized “ZOOM dysmorphia,” with majority agreement that virtual communications platforms during the COVID-19 pandemic had been associated with an increase in patient concerns about under-eye hollows (91%), dark eye circles (80%), and looking tired (60%).^18^ An absence of eye bags and a high, smooth lid-cheek junction were regarded as the most important contributors to the youthful eye, followed by brow position, hollowing, and infraorbital skin quality (Supplemental Figure 1, available at www.aestheticsurgeryjournal.com). Taking these factors into consideration, survey respondents unanimously agreed that midcheek volume was critical when treating tear-trough depression. Only 26% of respondents reported employing a specific classification system for assessing tear-trough depression, with the majority relying on clinical assessment and serial photography instead. When scales were utilized, the most frequently cited was Hirmand's classification, followed by the Barton tear-trough grading system.^19,20^

### Best Practice Approach to Patient Assessment

**Figure 1.**
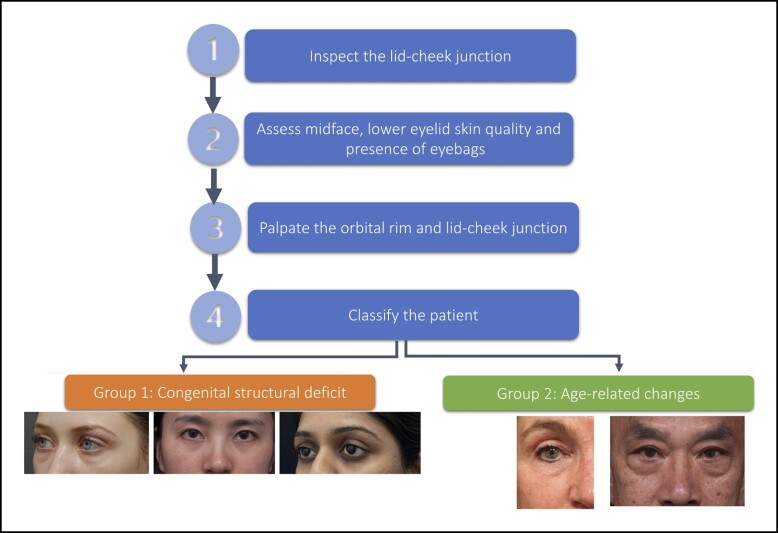
Lower eyelid dark circles: stepwise assessment framework.

A variety of factors can contribute to the appearance of lower eyelid dark shadows—bone structure, midface soft tissues (fat, muscles, ligaments), and skin quality. Determining which factors are most prevalent provides information to guide treatment approaches and counsel patients about expectations, both of which contribute to the development of an appropriate treatment plan. Figure 1 displays a proposed stepwise approach to assessment.

### Step 1: Inspect the Lid-Cheek Junction

**Figure 2.**
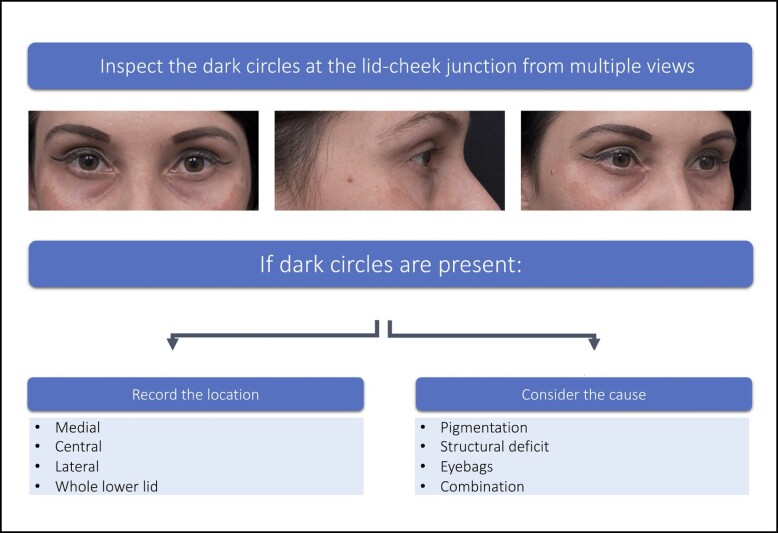
Inspection of the lid-cheek junction. Patient images reproduced from Liew and DeSilva (with permission from Georg Thieme Verlag KG).^15^

In patients who present seeking correction of dark circles, physical examination of the lower lid-cheek junction should be conducted with the patient sitting upright directly in front of the examiner at eye level (Figure 2).

After an initial general inspection of the overall facial aesthetics, a more detailed inspection of the shadowing at the lid-cheek junction should be undertaken in a well-lit room in multiple views (frontal, oblique, and lateral). The lid-cheek junction can be divided into 3 segments (Supplemental Figure 2, available at www.aestheticsurgeryjournal.com). If present, the location of the dark circles should be recorded (Table 1).

**Table 1.** Functional Classification of Lid-Cheek Junction

Whole lower eyelid-cheek junction: Surface landmark bounded by the tear trough ligament and orbicularis retaining ligament	
Medial lid-cheek junction	Inner third: Area of the lower eyelid extending inferolaterally from the medial canthus and to a point where the vertical line from the medial limbus meets the eyelid
Central lid-cheek junction	Middle third: Area extending from where the vertical line from medial limbus bisects the lower eyelid to the point where a vertical line from the lateral canthal angle meets the lower eyelid
Lateral lid-cheek junction	Outer third: Area extending from the point where a vertical line from the lateral canthal angle meets the lower eyelid to the lateral limit of the lower eyelid

Further inspection should then be undertaken to determine if the dark circles are due to skin pigmentation, structural deficits, the presence of eye bags, or a combination of these. Lighting and patient position can highlight specific features, and both play a key role in determining the cause of dark circles (Table 2).

**Table 2.** Importance of Lighting and Position

Potential underlying cause	Practical advice
Pigmentation	To accentuate pigmentation: Use a bright light on the skin and stretch the lower eyelid skin. The presence of skin pigmentation will be clearly visible. Equally those with transmitted pigmentation of underlying vascularity of the orbicularis oculi from overlying thin eyelid skin can be determined.
Structural deficits	To accentuate dark circles caused by structural deficits and/or eye bags: Use an overhead light directly above the patient or dim the ambient light.
Eye bags	To accentuate the presence and contribution of eye bags to dark circle: ask the patient to look up, without changing their facial position. The protuberance of eye bags will increase the prominence of dark circles.

### Step 2: Assess Midface, Lower Eyelid Fat Pads, and Skin Quality

**Figure 3.**
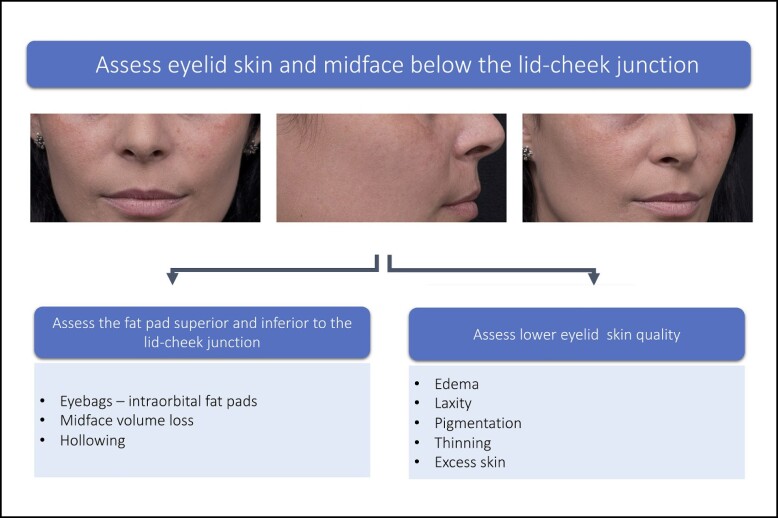
Assess midface and lower eyelid skin quality. Patient images reproduced from Liew and DeSilva (with permission from Georg Thieme Verlag KG).^15^

Tear trough inspection should include assessment of the fat pad superior and inferior to the lid cheek junction. Superiorly, excess infraorbital fat pads can contribute to the presence or perceived presence of dark circles (Table 2). Assessment should also include the midface below the lid-cheek junction (Figure 3) to determine any midface volume deficit. Assessment of volume deficit should focus specifically on the medial suborbicularis oculi fat (SOOF) and deep medial cheek fat (DMCF) that could be contributing to the demarcation of the lid-cheek junction and the appearance of the dark circles. Liew and DaSilva provide a detailed description on the rigid nature of the tear trough ligamental attachment medially and the extensibility of the central part of the orbital retaining ligament of the lid-cheek junction.^15^

Excess skin, thinness of skin, and skin crepiness have been identified as factors that may negatively impact the results of filler treatments.^6,10,15^ The quality of the lower eyelid skin should be assessed to determine if skin pigmentation is present and contributes to the dark circles. Evaluation of the lower eyelid should encompass objective and subjective measures of skin quality, with validated scales when available (Table 3).

**Table 3.** Skin Quality Factors That Contribute to Dark Eye Circles

Skin quality factors
Visually assess the patient for presence/absence of skin quality issues that might compound the appearance of dark circles: Thin skin Laxity Excess skin Edema Wrinkles^a^ Pigmentation^b^

^a^Validated objective scale, eg, Glogau scale.^21^  ^b^Validated photonumeric assessment scales are available to determine the severity of hyperpigmentation which also account for ethnic differences in skin tone.^22-25^

### Step 3: Palpate the Orbital Rim and Lid-Cheek Junction

**Figure 4.**
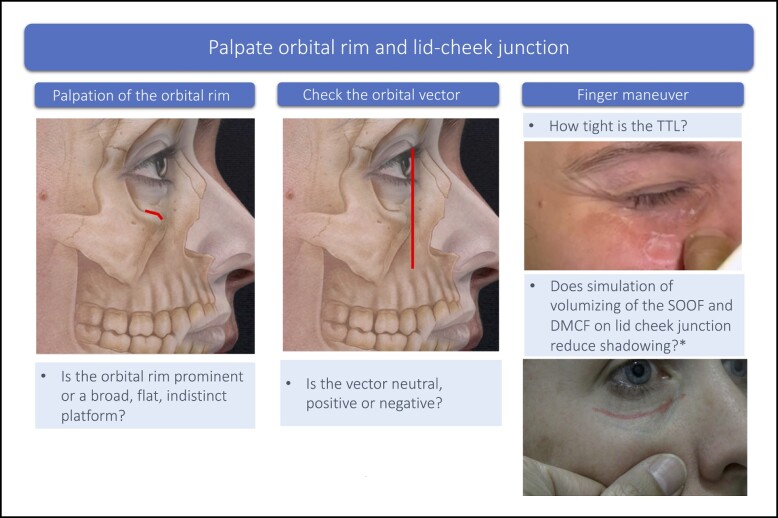
Palpation of the orbital rim and lid-cheek junction. Underlying bone tissue image, sourced from Mendelson and Wong.^27^ *Finger on midface pushing cephalad toward the lid-cheek junction, simulating the effect on volumizing the medial SOOF on lid-cheek junction. DMCF, deep medial cheek fat; SOOF, suborbicularis oculi fat; TTL, tear trough ligament.

Palpation of the orbital rim is conducted to determine if there is any underlying structural deficit, if so whether it is limited to the orbital rim, part of it or involving the whole orbital rim, and if it involves the maxilla, which is prevalent among East Asian patients.^26^ The orbital vector is determined with the patient in a profile position (Figure 4).^27^ It refers to the relative position of the globe to that of the supporting bony orbital floor, which is formed mainly by the maxillary bone.^28,29^ Patients with negative orbital vectors tend to present early with dark circles due to orbital fat prominence. With the patient gazing forward, place a finger on the anterior surface of the orbital rim and determine the relative position of the apex of the cornea to the orbital rim by dropping an imaginary line from apex of the cornea to the orbital rim. The vector is described as positive when the orbital rim is in front of the corneal apex, negative when the orbital rim is behind the cornea and neutral when it is in a vertical plane.^17^ Kato et al provide schematic images showing the relationship between the facial vector and eye bags.^17^

If tear-trough depression is present, gentle pulling of the skin from the underlying bone with finger and thumb will give an indication of the tightness of the tear trough ligament (TTL). A tight TTL is indicative of a difficult case for optimal correction. In this case, the patient needs to be counseled about realistic expectations from filler treatment.

In patients with volume loss to the midface, finger maneuvers can simulate the effect of volumizing of the medial SOOF and DMCF on the lid-cheek junction. To help determine the extent to which midfacial volume loss is contributing to the dark circles, gently push the midfacial tissue just caudal to the medial SOOF in a cephalad direction toward the lid-cheek junction. This will simulate the effect of medial SOOF volumization and visually highlight the improvement of the lid-cheek junction.^15^

### Step 4: Patient Classification

Based on the assessment process above, patients with dark circles associated with some degree of depression or hollowing of the lid-cheek junction can be classified into 2 broad groups (Table 4). Group 1 comprises patients with dark circle depression that is due to an anatomical structural deficit from the underlying bone. Because this is not associated with aging, these cases tend to be younger patients in their early adulthood. They are not associated with midfacial volume loss. The other, larger group includes those with lid-cheek junction pathology secondary to the aging process, with age-associated maxillary retrusion, deep fat pad volume loss, and the associated degeneration changes of the overlying skin, muscle, and orbital retaining ligament and orbital septum.^15^

**Table 4.** Classification of Patients Presenting With Dark Circles

Group	Characteristics
Group 1	Congenital structural bony deficiencies of: Maxilla Orbital margin/rim
Group 2	Age-related changes (bony recession and soft tissue deflation) with: Tear trough and infraorbital hollow (Group 2a) Prominent eye bags (Group 2b)

Adapted from Liew and De Dilva, with permission.^15^

### Group 1: Congenital Structural Deficit of the Underlying Maxilla

**Figure 5.**
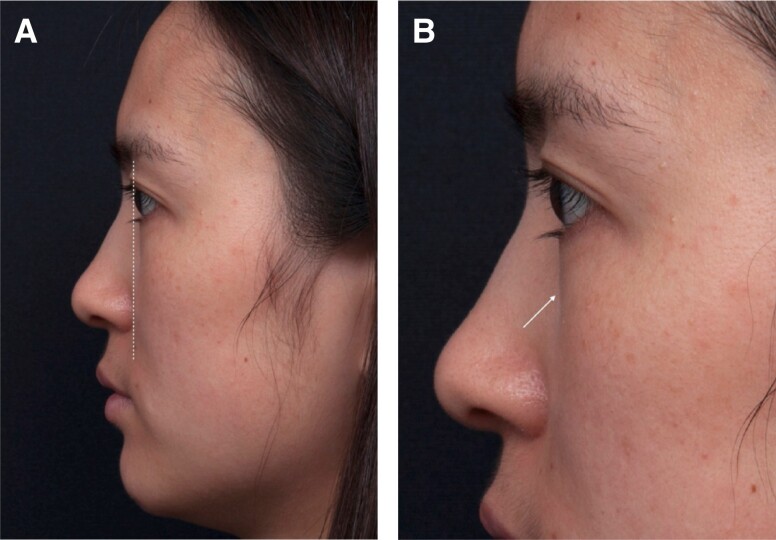
Classification Group 1b. A 25-year-old female displaying typical features including a negative orbital vector (A) and pseudo eye bags (B, arrow).

This group of patients tends to present complaining of dark circles early in adulthood or in the late teens. Within this group there are 3 subvariants:

- 1a: The structural deficit is confined to the medial orbital rim of the maxilla. Patients tend to present in their early adulthood with deep tear-trough depression associated with a minimal degree of extension laterally and minimal to no volume loss of the midface (Supplemental Figure 3, available at www.aestheticsurgeryjournal.com).
- 1b: The entire maxilla is retruded and there is a negative orbital vector (Figure 5A). This group of patients tends to present early complaining of dark circles or eye bags. Anatomically, the retrusion of the maxilla creates a “short orbital floor” for the size of the globe and the orbital content. This leads to a spillage of the orbital fat beyond the lower orbital rim rather than excessive fat pad, causing “pseudo eye bags” at an earlier age (Figure 5B). The same anatomical retrusion of the maxilla also creates a recessed alar base with deep nasolabial retrusion at an early age. Group 1b is common among patients of Northeast and Southeast Asian ethnicity.
- 1c: The structural deficit is confined mainly to the entire maxillary orbital rim. Palpation of the lower orbital rim displays a recessive orbital rim posteriorly beyond that of the body of the maxilla. The lower eyelid in this group tends to be short and recline further posteriorly as it approaches the lid-cheek junction. This eyelid configuration creates a distinct eye appearance of large prominent eyes with excessive scleral show (Supplemental Figure 4, available at www.aestheticsurgeryjournal.com). Group 1c is common among patients of Southern Asian, Middle Eastern, Turkish, and Iranian descents.

### Group 2: Age-related Changes

This group of patients tends to present from the late 20s to the early 30s onward, complaining of looking tired and of lower eyelid dark circles. Group 2 constitutes the main bulk of patients seeking treatment for dark circles. This group also encompasses the original Group 1 cohort whose structural deficits are now compounded by the aging process and its sequelae. In this case, the lower eyelid appearance is associated with various degrees of midfacial aging. Within this group there are 2 broad categories:

- 2a: There is typically recession and hollowing along the lid-cheek junction, and the hollowed appearance may extend across the entire lid-cheek junction (Supplemental Figure 5).
- 2b: These patients present with predominantly lower eye bag formation associated with a varying degree of lid-cheek junction hollowing (Supplemental Figure S5, available at www.aestheticsurgeryjournal.com).

### Practical Tips for Treatment Planning

**Figure 6.**
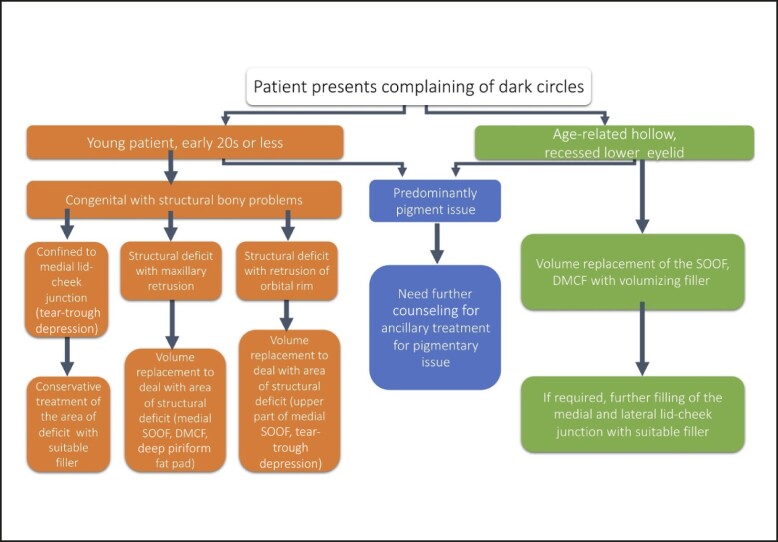
Patient characteristics and treatment planning. DMCF, deep medial cheek fat; SOOF, suborbicularis oculi fat.

**Figure 7.**
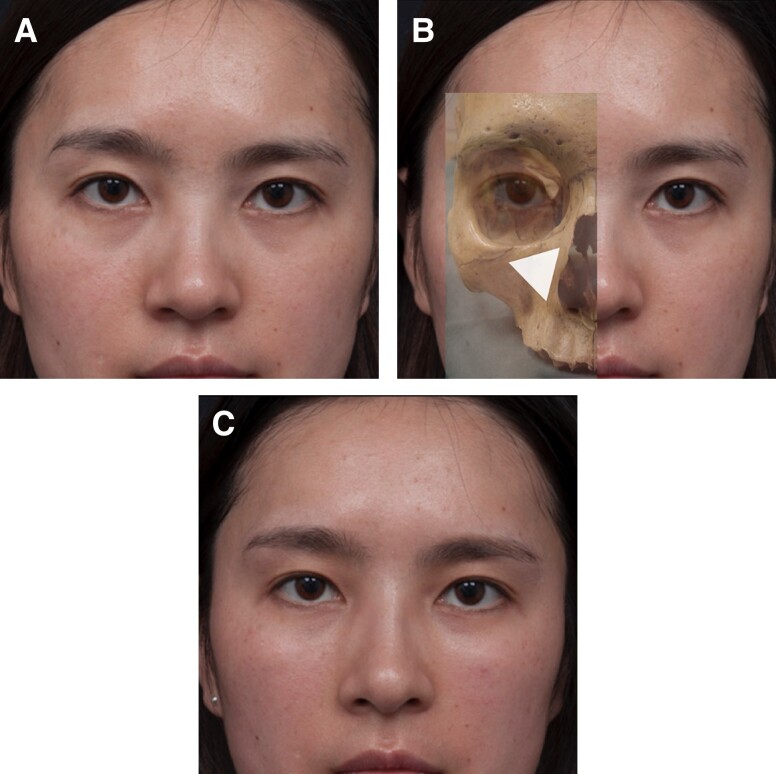
(A) The same Group 1b 25-year-old female shown in Figure 5 presented with dark lower lid circles. (B) The lower eyelid dark circle is due to structural retrusion of the maxilla. The triangle shows the position of the underlying structural bony deficit and indicates the optimal placement of filler in the medial suborbicularis oculi fat pad and deep medial cheek fat pad to achieve correction. (C) Three months post injection with a small volume of soft hyaluronic acid filler, showing an elevation and effacement of the lid-cheek junction and improved appearance of dark circles without actual treatment of the lower eyelid region.

**Figure 8.**
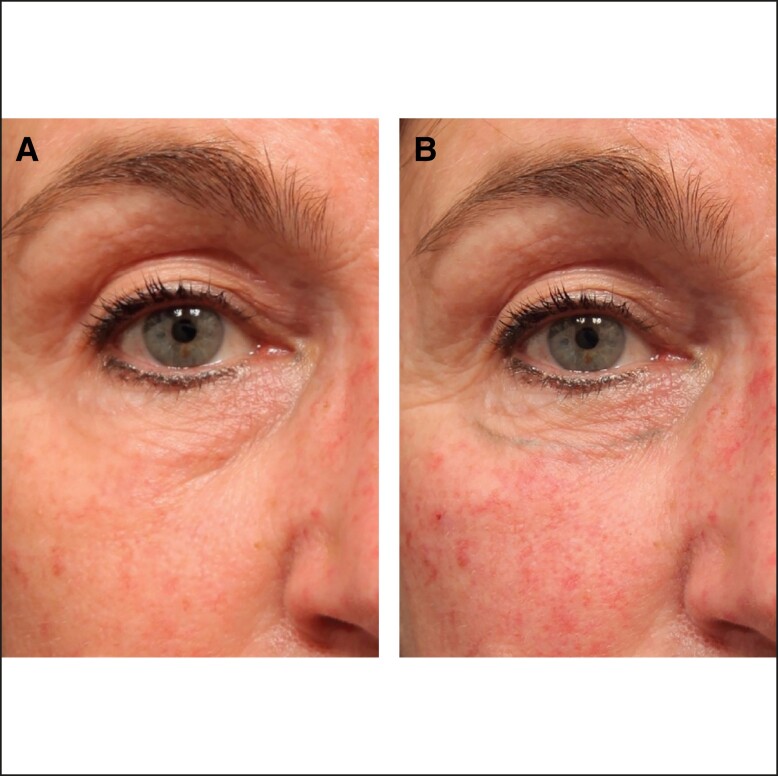
Classification Group 2a. (A) A 54-year-old female exhibiting depression across all segments of the lid-cheek junction. (B) The same patient immediately following filling of the medial suborbicularis oculi fat pad and deep medial cheek fat pad with filler, showing elevation and effacement of the central lid-cheek junction. Image reproduced from Liew and DeSilva, with permission from Georg Thieme Verlag KG.^15^

A detailed description of treatment for each of the above groups is beyond the scope of this article. In essence a more precise understanding of the appearance of the lid-cheek junction pathology, as described through the steps in our assessment framework, enables the clinician to optimize the most logical ways of treating each patient type (Figure 6). For example:

- Group 1a: Requires precise placement of the minimum amount of the least hygroscopic filler deep on the medial orbital rim, with the aid of stretching of those with a tight TTL to ensure adequate correction with the least amount of product and minimize potential swelling posttreatment.^26^
- Group 1b: Requires treatment to deal with the structural retrusion of the maxilla by placement of an appropriate volumizing filler in the medial SOOF, DMCF, and/or deep piriform fat pad. By assessing the patient (Figure 7A) and determining the underlying cause of the dark circles (Figure 7B), dark circle correction can be accomplished without the need to directly inject the tear trough (Figure 7C).
- Group 1c: Requires filler placed just below the orbicularis retaining ligament within the upper portion of the medial SOOF and a minute amount of filler placed judiciously on the bony level at the tear-trough depression, where it can be applied as a filler strut to augment the retruded orbital rim and correct the hollowness.
- Group 2a: Requires replenishment of volume loss of the medial SOOF and DMCF to see the extent of improvement of the lid-cheek junction (Figure 8). Once that is achieved, consultation with the patient should be undertaken to decide if any of the residual deficit in the tear-trough depression needs to be dealt with. If this is determined necessary, then additional smaller volumes of filler can be injected directly into the medial and lateral lid-cheek junction.
- Group 2b: Patients in this subgroup are candidates for surgery, and therefore treatment is beyond the scope of this article.

## DISCUSSION

In this article, the phrase “dark eye circles” was intentionally utilized because this is the lay term coined by patients as they seek treatment. Equally, this term helps clarify to practitioners the number of etiological factors that can present as common complaints that are not amenable to HA filler treatment.

HA fillers are an effective solution for the correction of tear-trough deformities.^30,31^ Despite having been available for many years, injecting HA fillers in the tear trough and the lower eyelid is technically challenging and associated with a high incidence of posttreatment complications.^6,7,16,32,33^ Why are we still seeing these problems and what can be done to minimize or avoid them?

The problem is directly related to the complex anatomy of the tear-trough area. The anatomy of the tear trough and lid-cheek junction has been previously reviewed.^15^ Due to the tight compartmental nature of this region, there is limited space within which to inject filler. If large volumes are injected, they can saturate the deeper muscle layer and be dispersed into the more mobile orbicularis muscle layer. Common complications, both early and late, associated with lower eyelid HA filler injections such as swelling, puffiness, Tyndall effect, and sausage-shaped lumps almost always appear on the medial lid-cheek junction as the result of too much filler being placed directly in 1 treatment, or repeatedly over time, in the tear trough.

We believe a proper assessment process can guide diagnosis, which in turn underpins appropriate patient selection, choice of management technique, and optimal outcomes. Several classification scales are available to help evaluate tear-trough deformity and guide treatment selection.^19,20,34-36^ Our practitioner survey highlighted a limited use of classification systems and an increased reliance on clinical assessment. However, comprehensive guidance on clinical assessment of dark eye circles is limited. Previous work supports a functional anatomical classification of the lid-cheek junction for soft tissue filler treatment.^15^ This classification has established a simplified clinical approach enabling categorization of patients presenting with dark eye circles into 2 broad groups—those with tear-trough depression due to underlying congenital bony structural deficits (Group 1) and those with age-related changes (Group 2).^15^ Here we provide a practical application of this classification system for a clinical setting. Through stepwise, detailed examination practitioners can assess patients presenting with lower eyelid dark circles and stratify them to guide a more strategic approach to treatment.

Palpation of the orbital rim is a crucial assessment step. It is important to determine if there are congenital abnormalities predisposing the patient to under-eye hollowness, particularly in younger patients in whom age-related changes do not yet apply.^37^ While ethnic differences in orbital rim morphology and depth of the orbital floor should be accounted for, it is established that filler in 1 area can enhance other areas without direct injection.^14,15,38-41^ Reflation of the SOOF and DMCF with HA filler can improve the appearance of the central part of the lid-cheek junction without requiring direct treatment of the lid-cheek junction itself. Foregoing the need for direct HA filler injection into the tear trough negates the risks of posttreatment complications due to overfilling in this area.

During initial physical inspection of the patient, the authors recommend strategic lighting and assessment from multiple views to provide helpful insights about the various underlying pathological and anatomical causes of shadowing. Overhead lighting worsens the appearance of dark eye circles, while direct lighting may mask them. Turning down the light intensity in the room helps to reveal the shadows, and looking at the oblique view helps to better visualize the extent of any infraorbital hollowing.

Three key contributors of skin quality to dark eye circles have been identified; lower eyelid skin thickness, the number and/or thickness of under-eye capillaries, and hyperpigmentation.^22^ The lower eyelid skin is extremely thin.^42^ It is also inelastic due to lower levels of collagen, elastin, and glycosaminoglycans than are observed in other areas of the face, and dry due to the presence of few sebaceous glands.^43^ Thinner skin contributes to the appearance of dark circles because it increases the likelihood that underlying pigmentation and dilated vessels are visible.^22^ This is further compounded by the aging process, during which loss of elasticity leads to folding and redundancy (dermatochalasis) with associated increases in edema, laxity, and sagging that accentuate the appearance of dark circles, and the appearance of wrinkles that accentuate the extent of fat prolapse and the depth of the visible grooves.^43-45^ Careful lighting and stretching of the skin allow a detailed inspection of the eyelid skin quality and can confirm or rule out pigmentation. Pigmentation does not contribute directly to the depth of the tear trough; rather it creates an illusion of depth.^45^ Pigmentation of the lower eyelid skin is relatively common as part of the presentation of dark circles, but will not be improved by HA filler injection.^44^ Irrespective of whether a patient falls into functional classification Group 1 or Group 2, if pigmentation is a contributory cause of their dark circles counseling on outcomes is paramount. Patients should be made aware of this and offered additional treatment options when necessary and appropriate.

This work has been underpinned by literature and expert opinion. The literature provides ample guidance on anatomy, injection technique, and management of posttreatment complications.^6-8,10-16^ But practical guidance on patient assessment is limited. The practitioner survey highlighted a preference for clinical assessment over formal classification systems, which in turn drove the development of an assessment algorithm. However, this research was undertaken in a selective group that could have introduced opinion bias. Although we do not believe this limitation to have resulted in any significant negative impact, future refinement might come from the application of the algorithm in a more diverse population. Formal further study is not necessary because the algorithm is not intended to provide a formal validated scale. However, it is our hope that in the future it can be incorporated into educational meetings at which attendees can be taught how to use the algorithm and apply it in their day-to-day practice.

## CONCLUSIONS

The utilization of HA fillers for dark eye circles is popular but challenging and associated with a high rate of complications. We present a comprehensive assessment framework that combines inspection, assessment, and palpation. The overall aim is to encourage practitioners to pause and think before they treat, rather than simply treating the problem location. Careful step-by-step assessment, conducted with judicious lighting, can help to identify patients in whom dark eye circles may be improved without the need to directly inject HA filler into the tear trough. Correct patient classification can facilitate individualized treatment planning and optimize treatment outcomes while avoiding the known challenges associated with treating this area.

## Supplemental Material

This article contains supplemental material located online at www.aestheticsurgeryjournal.com.

## Supplementary Material

sjae058_Supplementary_Data
