# Supplementary material for: Lower Eyelid Dark Circles (Tear Trough and Lid-Cheek Junction): A Stepwise Assessment Framework
Source: Aesthet Surg J. 2024 Mar 15;44(7):NP476–85. doi: 10.1093/asj/sjae058 (PMC11177555; doi:10.1093/asj/sjae058)
Supplement: sjae058_Supplementary_Data [file sjae058_supplementary_data.zip › Appendix_Periorbital_Survey_ASJ_23-1139_final.pdf]

First Impressions Scientific Exchange Practitioner Survey

SURVEY QUESTIONS

1) What is your primary medical specialty?

[Select one only]

- Dermatologist
- Surgeon (Plastic/Cosmetic)
- Oral maxillofacial surgeon
- Oculoplastic surgeon
- Cosmetic physician
- Primary care physician/General Practitioner
- Registered Nurse/Nurse Practitioner
- Other

2) How many years have you been practicing aesthetic medicine?

[Select one only]

- <12 months
- 1-3 years
- 3-5 years
- 5-10 years
- > 10 years

3) Please rank the following in order of importance to a person's overall aesthetic "First impression"

[Assign 1 to the most important, 2 to the next important etc]

- Facial profile
- Facial shape
- Facial contours
- Facial proportions
- Facial symmetry
- Eyes/eyelid aesthetics
- Facial expressions (static/dynamic lines [emotional expression])
- Smile/lip aesthetics
- Skin quality/evenness of tone

Skin texture
Skin firmness

4)	Which of the following assessment instruments do you use in your practice?	[Select all that apply]<br>Facial Assessment Scale<br>Global facial aesthetic appearance scale<br>Patient assessment scale<br>Skin Quality Assessment<br>Global aesthetic improvement scale<br>Patient satisfaction questionnaire<br>Photo-numeric scales<br>Visia or similar skin grading software<br>Serial clinical photography<br>Quality of life assessments<br>Other<br>None of the above
5)	Does beauty affect a person's quality of life?	[Select one only]<br>Yes<br>No<br>No opinion
6)	The COVID-19 pandemic has led to an increase in mask-wearing and video-conferencing calls in which patients are not able to apply photographic filters. Have you noticed a change in the types of requests for cosmetic procedures compared to before the COVID-19 pandemic?	No<br>Yes<br>If yes, which of the following have your patients been concerned about:<br>[Select all that apply]<br>Problems with animation<br>Expression lines<br>Lower face wrinkles<br>Upper face wrinkles<br>Asymmetry<br>Facial dark spots<br>Dark eye circles<br>Under eye hollowness<br>Looking tired<br>Jowls<br>Neck ageing<br>Facial volume loss

7) To what extent do each of the following attributes contribute to youthful eyes?

[Mark the most appropriate number on the scale; 0 = no contribution, 10 = maximum contribution]

- Brow position
- Shape of the eyebrow
- Skin quality of eyelid skin
- Volume of the upper eyelid
- Shape of the eye aperture
- Upper eyelid position
- Lower eyelid position
- Volume of eye bags
- Pigmentation of the eyelid skin
- Depth and position of the tear trough and lid-cheek junction
- Presence of skin eyelid skin oedema
- Presence of orbital hollowing

8) Do you account for ethnic differences when defining a beautiful eye?

[Select one only]

- Yes
- No
- No opinion

9) Which of the following can impact the outcome treatment of tear trough depression?

[Select all that apply]

- Periorbital hyperpigmentation
- Fat pad prolapse
- Lower eyelid/malar oedema
- Lymphatic sufficiency
- Excess lower eyelid skin
- Depth of tear trough deformity
- Lower eyelid skin discolouration
- Mid-cheek volume
- Loss of support for ligaments
- Patient expectations

10) When treating the tear trough depression, do you treat the midface first?

[Select all that apply]

- Always
- Never
- If the patient requests for it
- Only if deficient
- Most of the time

11) Do you use a particular classification system for assessing tear trough depression?

[Select one only]

- No
- Yes

If yes, please specify which classification system you use

12) What is your preferred choice of injection method when using HA filler for tear trough depression?

[Select one only]

- Cannula
- Needle
- It depends on the individual patient
- Neither do not use HA filler for tear trough
- Other

13) Please rank the following injection sites/techniques for the use of HA filler for tear trough depression in order of preference

[Assign 1 to the most preferred, 2 to the next preferred etc]

- Supra-periosteal
- Superficial under the skin
- Sandwiched technique (superficial and deep pre-periosteal)
- Do not use HA filler for tear trough
- Do not treat
- No preference

14) Please rank the following treatments for the lower eyelid skin quality (including wrinkles) in order of preference

[Assign 1 to the most preferred, 2 to the next preferred etc]

- Cosmeceuticals
- Chemical peels
- Laser
- Microagulants (Plasma pen)
- Fillers
- Neuromodulators
- Radiofrequency devices
- Micro-needling
- Platelet-rich plasma
- PDO monothreads
- Bio-stimulants
- Do not treat
- No preference

15) Please rank the following treatments for periorbital hyperpigmentation in order of preference

[Assign 1 to the most preferred, 2 to the next preferred etc]

- Topical depigmenting agents
- Lasers/light device
- Fillers
- Chemical peels

Carboxytherapy
Platelet rich plasma injection
Micro-needling
Do not treat
No preference
