# Supplementary material for: Lower Eyelid Dark Circles (Tear Trough and Lid-Cheek Junction): A Stepwise Assessment Framework
Source: Aesthet Surg J. 2024 Mar 15;44(7):NP476–85. doi: 10.1093/asj/sjae058 (PMC11177555; doi:10.1093/asj/sjae058)
Supplement: sjae058_Supplementary_Data [file sjae058_supplementary_data.zip › SuppTable1_ASJ_23-1139_final.docx]

**Supplemental Table 1**

Practitioner Profile: Demographics

| **Country of residence** | **N (%)** |
| --- | --- |
| Mexico | 1 (2.5%) |
| Brazil | 1 (2.5%) |
| Sweden | 1 (2.5%) |
| Indonesia | 1 (2.5%) |
| Philippines | 1 (2.5%) |
| New Zealand | 2 (5.0%) |
| Hong Kong | 2 (5.0%) |
| Korea | 2 (5.0%) |
| China | 2 (5.0%) |
| Singapore | 3 (7.7%) |
| Thailand | 3 (7.7%) |
| Taiwan | 3 (7.7%) |
| Australia | 18 (46.0%) |

* 39/40 surveys returned. Not all surveys were fully completed; no imputation for missing data.
