# Supplementary figures and images for: Lower Eyelid Dark Circles (Tear Trough and Lid-Cheek Junction): A Stepwise Assessment Framework
Source: Aesthet Surg J. 2024 Mar 15;44(7):NP476–85. doi: 10.1093/asj/sjae058 (PMC11177555; doi:10.1093/asj/sjae058)

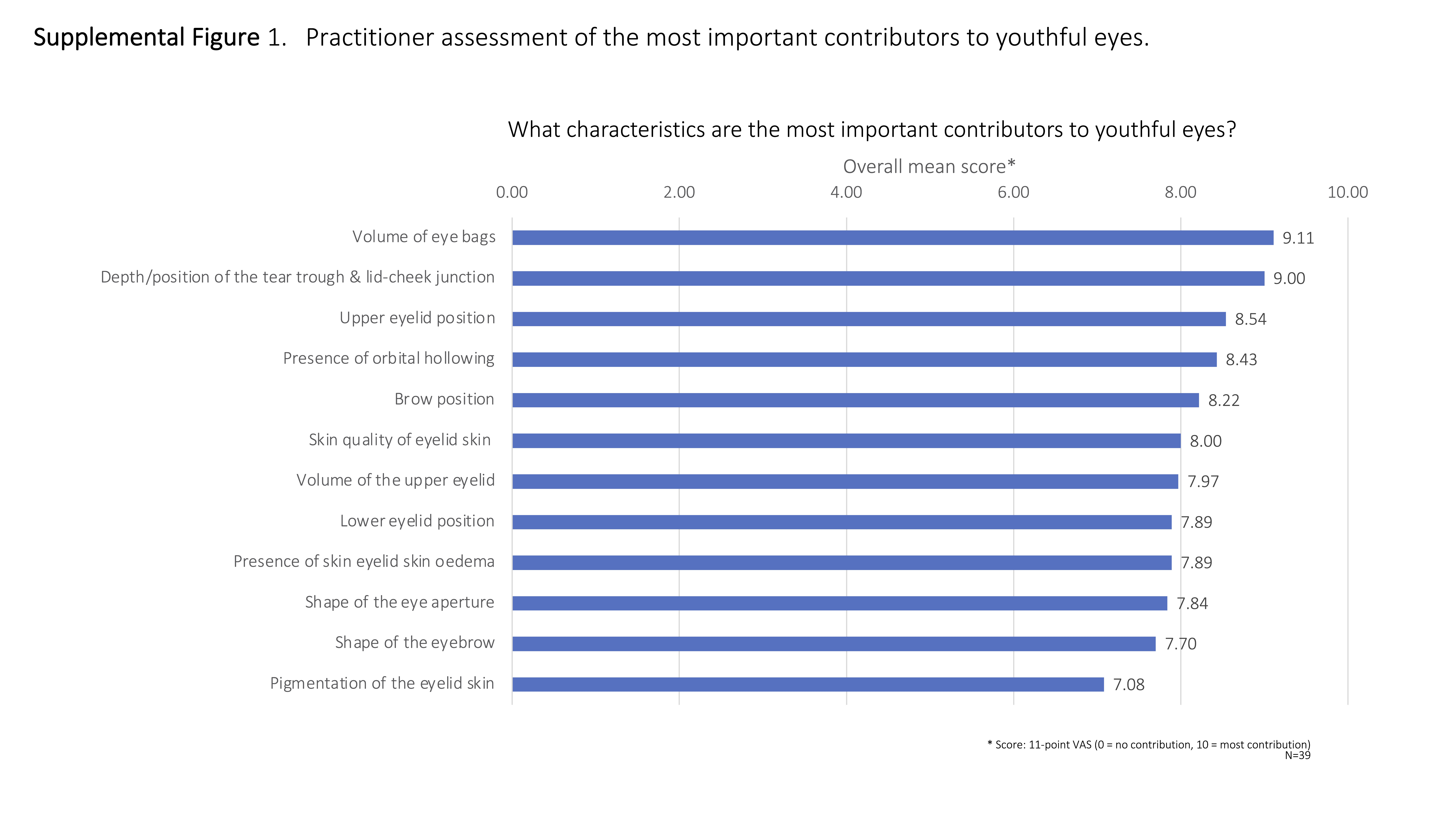

Supplement: sjae058_Supplementary_Data [file sjae058_supplementary_data.zip › SuppFig1_ASJ_23-1139.png]

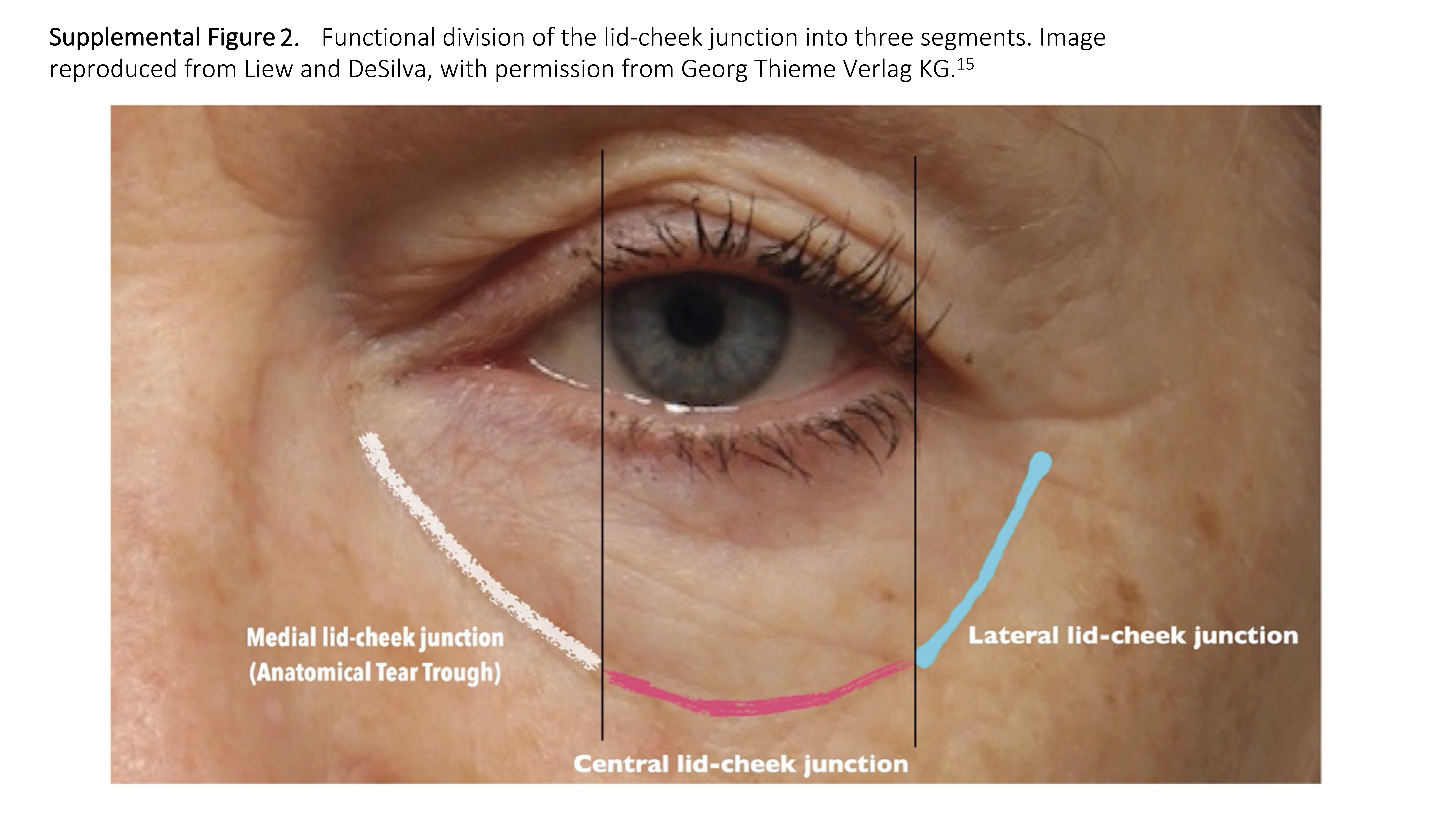

Supplement: sjae058_Supplementary_Data [file sjae058_supplementary_data.zip › SuppFig2_ASJ_23-1139.png]

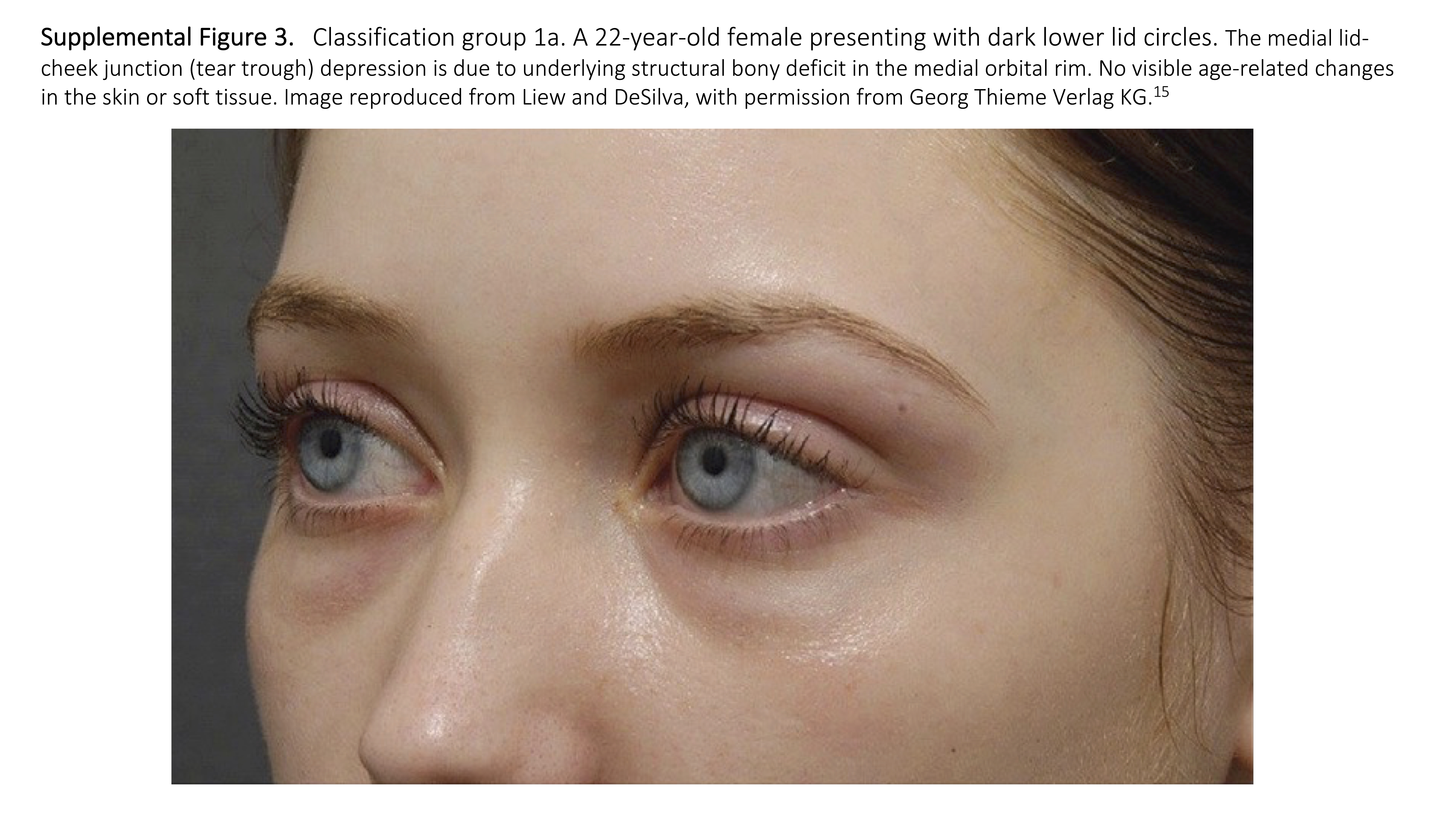

Supplement: sjae058_Supplementary_Data [file sjae058_supplementary_data.zip › SuppFig3_ASJ_23-1139.png]

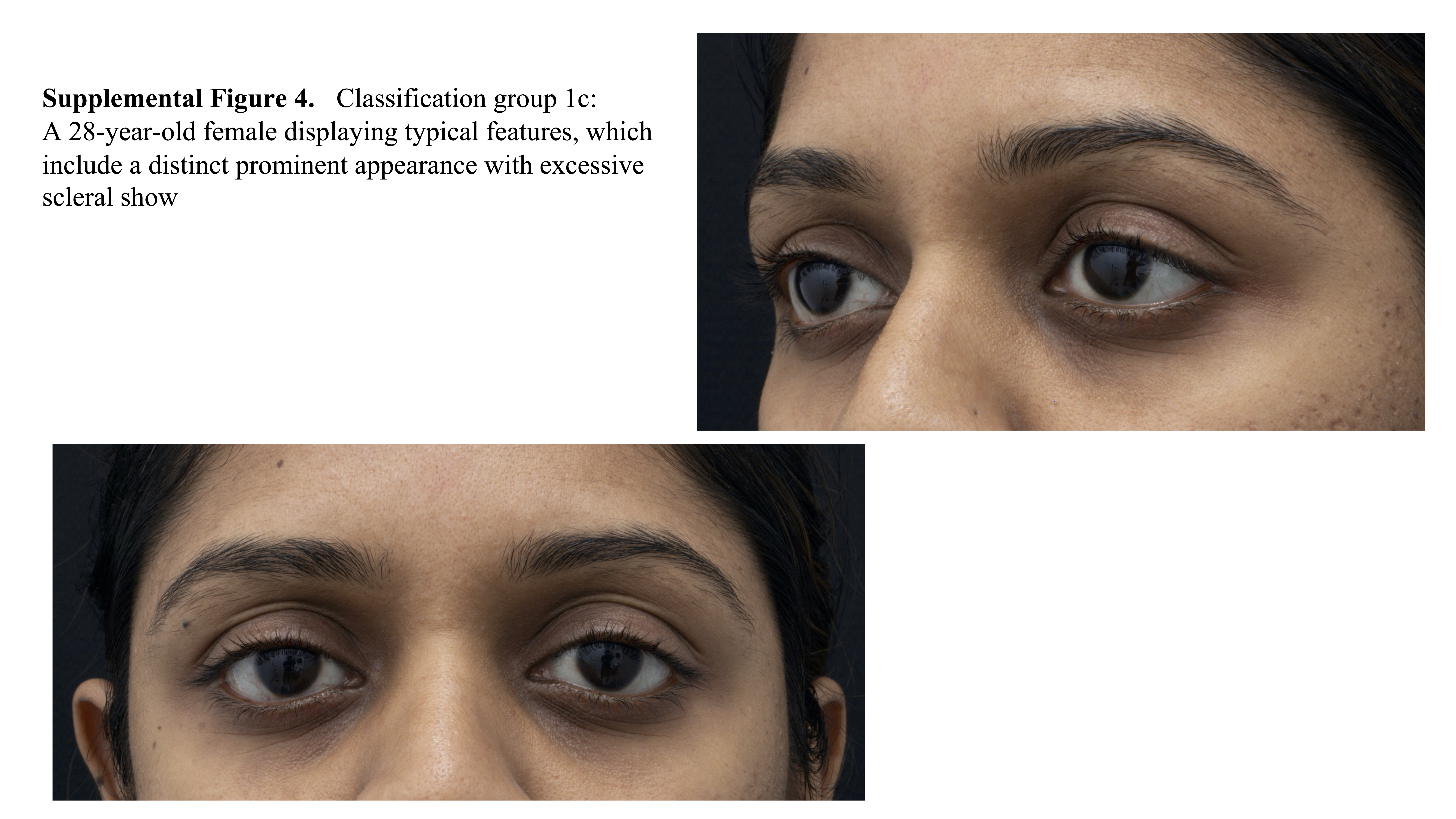

Supplement: sjae058_Supplementary_Data [file sjae058_supplementary_data.zip › SuppFig4_ASJ_23-1139.png]

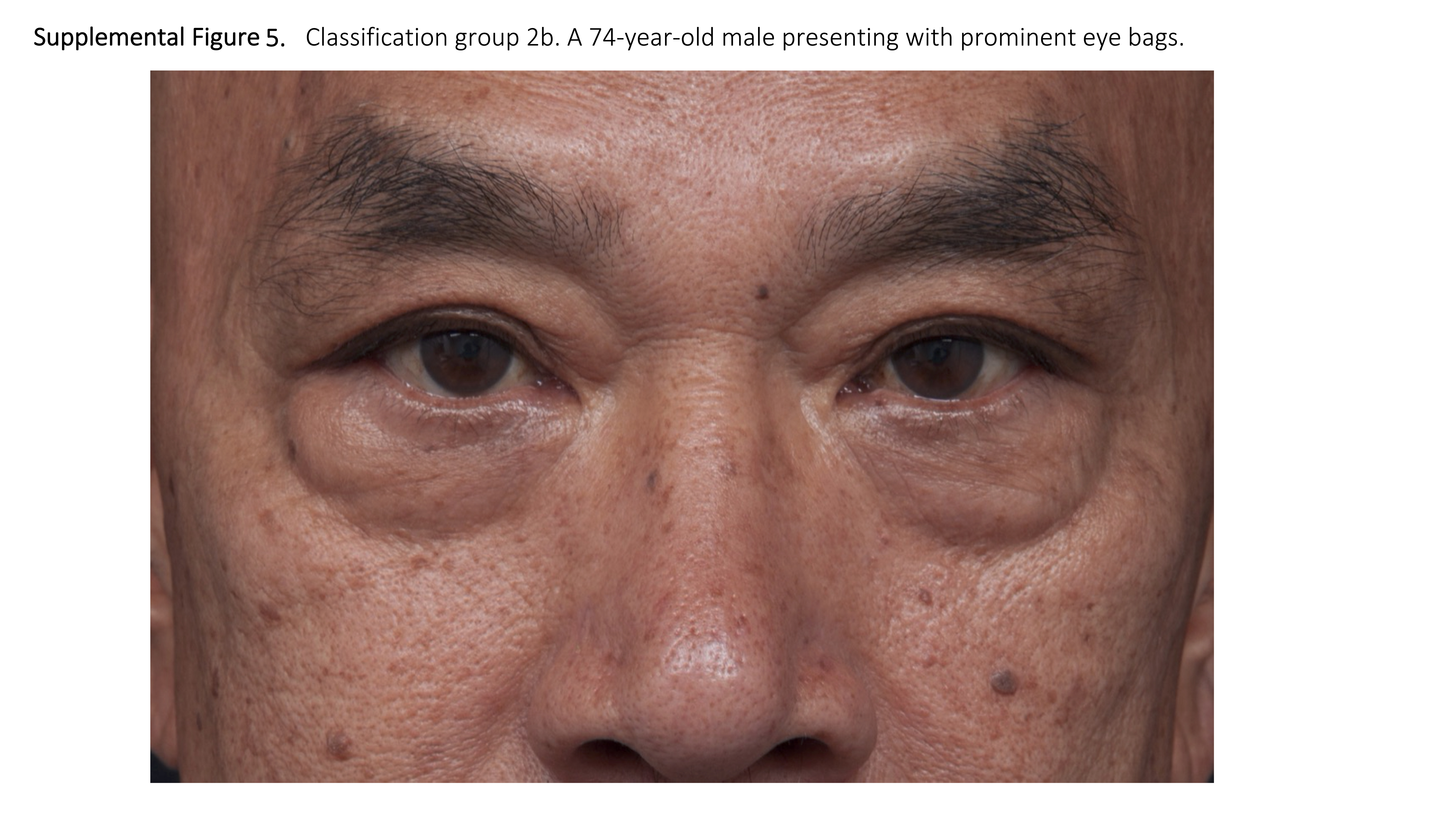

Supplement: sjae058_Supplementary_Data [file sjae058_supplementary_data.zip › SuppFig5_ASJ_23-1139.png]
